# Cochlear synaptic vulnerability scales with pathological noise intensity in a noise-induced hearing loss model

**DOI:** 10.1007/s13258-026-01748-w

**Published:** 2026-03-04

**Authors:** Byeonghyeon Lee, Tae-Jun Kwon, Hyun Tae Kim, Un-Kyung Kim

**Affiliations:** 1 New Drug Development Center, Daegu-Gyeongbuk Medical Innovation Foundation (K-MEDI hub), 41061 Daegu, Republic of Korea; 2 Preclinical Research Center, Daegu-Gyeongbuk Medical Innovation Foundation (K-MEDI hub), 41061 Daegu, Republic of Korea; 3 Bio-Medical Research Institute, Kyungpook National University Hospital, 41940 Daegu, Republic of Korea; 4 Department of Biology, College of Natural Sciences, Kyungpook National University, 41566 Daegu, Republic of Korea; 5 School of Life Sciences, BK21 Plus KNU Creative BioResearch Group, Kyungpook National University, 41566 Daegu, Republic of Korea; 6 Adcanced Bio-Resource Research Center, Kyungpook National University, 41566 Daegu, Republic of Korea

**Keywords:** Noise-induced hearing loss, Cochlear synaptopathy, Synaptic ribbon, Functional synapse pairing, Mouse model

## Abstract

**Background:**

Noise-induced hearing loss (NIHL) has been traditionally attributed to outer hair cell (OHC) damage, but synaptic injury at inner hair cell (IHC)–spiral ganglion neuron (SGN) junctions may better explain persistent functional deficits.

**Objective:**

To test whether IHC ribbon loss and functional synapse loss scale with pathological noise intensity and to define their longitudinal relationship to hearing function.

**Methods:**

A rodent NIHL model was exposed to 100 dB SPL for 2 h or 110 dB SPL for 4 h. Cochleae were immunolabeled for CtBP2 (presynaptic ribbons) and GluA2 (postsynaptic AMPA receptor puncta). Putative functional synapses were quantified as closely apposed CtBP2/GluA2 pairs. Hearing function was assessed longitudinally and correlated with synaptic measures.

**Results:**

Both exposure conditions produced graded reductions in IHC ribbon counts and CtBP2/GluA2 pairs, with greater losses after 110 dB/4 h. The number of CtBP2/GluA2 apposed pairs closely tracked preserved hearing function, providing a stronger association with functional outcome than ribbon counts alone. Ribbon loss remained largely stable across post-exposure intervals, whereas functional synapse loss was disproportionately larger at early time points, consistent with transient pre–post misalignment. Over time, CtBP2/GluA2 pairing partially recovered toward levels predicted by surviving ribbons, suggesting re-alignment or reconnection of a subset of remaining synaptic elements.

**Conclusion:**

Pathological noise intensity drives exposure-dependent IHC synaptopathy in addition to OHC injury. Functional synapse counts (CtBP2/GluA2 pairs) sensitively reflect stored hearing function and indicate a potential post-exposure window for synaptic re-establishment.

## Introduction

Hearing loss is among the most widespread global health conditions and represents one of the most common sensory impairments in humans (Collaborators 2021; Wilson and Tucci 2021). For congenital sensorineural hearing loss, reported birth prevalence is approximately 1.2–1.7 per 1,000 live births (Choe et al. 2023; Yoshimura et al. 2024). The proportion of affected individuals rises with age: estimates increase to ~ 2.7 per 1,000 by 5 years of age, with a further increase to ~ 3.5 per 1,000 during adolescence (Korver et al. 2017). Because hearing loss spans a broad spectrum of etiologies and severity, its overall prevalence varies considerably across populations (Collaborators 2021; Yoshimura et al. 2024). Although it is not typically life-threatening, hearing loss can impose substantial educational, social, and occupational burdens, and these consequences may be amplified in settings with limited resources, low literacy, and reduced access to specialized support (Podury et al. 2023; Pryce et al. 2025). Unilateral or mild-to-moderate bilateral hearing loss is common in children and can be under detected early, leading to delayed identification and intervention (Alde et al. 2024). Mild or moderate deficits are often missed until school age, reflecting limitations in routine detection pathways.

Noise-induced hearing loss (NIHL) is a major form of acquired hearing loss and has been described as a leading cause after age-related loss in the United Kingdom (Chen and Su 2025). NIHL is classically associated with older adults (≥ 65 years), yet several reports indicate increasing prevalence among children and young adults as well (Shargorodsky et al. 2010). High-intensity sound exposures occur in occupational environments such as construction, mining, and aviation. Importantly, acoustic insults can differ in temporal profile: brief, high-level “impulse” exposures (e.g., gunfire) may drive rapid injury, whereas continuous exposures (e.g., machinery noise or loud music) often produce cumulative damage across time (Wong et al. 2013). In recent years, recreational exposures have gained prominence as a risk source, particularly in adolescents and young adults (Shargorodsky et al. 2010). Risk depends on the intensity–duration dose of exposure, and repeated high-level recreational listening has been associated with early auditory changes in multiple populations (Dillard et al. 2022).

Shifts in music consumption may contribute to these trends. Attendance at concerts or nightclubs—where sound levels are often high—can reinforce social norms that equate loudness with entertainment. In parallel, widespread use of personal listening devices and increasingly effective headphones can encourage users to raise volume to overcome ambient noise. Because environmental background levels in many urban settings are also elevated (traffic, construction, dense pedestrian activity), the combined effect may further increase exposure burden in daily life (McAlexander et al. 2015).

Several modifiable host factors have been associated with NIHL vulnerability, including smoking and comorbid conditions such as diabetes and cardiovascular disease (Chen and Su 2025; Xue et al. 2025). These risk factors overlap with features of metabolic syndrome, a clinical cluster often defined by the presence of multiple cardiometabolic abnormalities (e.g., central obesity, hypertriglyceridemia, hypertension, pro-thrombotic tendency, and insulin resistance). Metabolic syndrome is linked to markedly increased risk for type 2 diabetes and elevated risk for cardiovascular events over subsequent years (Kaur 2014). Such associations support the hypothesis that microvascular compromise and impaired cochlear perfusion may contribute to NIHL susceptibility. Non-modifiable influences—such as age, genetic background, and ancestry—have also been discussed in relation to NIHL risk (Campbell et al. 2023; Chen et al. 2022).

Clinically, high-level noise exposure can produce tinnitus and a transient elevation in hearing thresholds, often referred to as a temporary threshold shift, which may improve after the exposure ends (Chen and Su 2025; Couth et al. 2024). Repeated exposures—especially when recovery between episodes is incomplete—may ultimately lead to permanent threshold elevation. Audiometrically, NIHL frequently manifests as disproportionate impairment at higher frequencies, commonly with a “notch”-like pattern around the 3–6 kHz region, often most pronounced near 4 kHz.

Even at early clinical stages, loss in the high-frequency range can degrade speech perception in challenging environments, particularly when background noise is present. This problem may be most evident for higher-pitched voices (e.g., women and children). Damage is often exacerbated by exposures that combine high intensity with high-frequency content (Wong et al. 2013). With respect to music exposure, the literature has debated the magnitude of risk, and some discrepancies across studies have been attributed to differences in measurement sensitivity. For example, approaches that assess hearing thresholds across more finely sampled frequency steps (e.g., sweep-based or high-resolution methods) may detect subtle, narrow-band deficits that standard pure-tone audiometry can miss (Jafari et al. 2022; Xiong et al. 2019). In addition, a broad review of the literature has argued that music exposure is sufficiently linked to adverse auditory outcomes and that early symptoms—such as transient threshold changes, tinnitus, or reduced hearing sensitivity—may serve as warning indicators of increased NIHL risk (Zhao et al. 2010).

From a mechanistic perspective, the cochlea’s sensory epithelium (the organ of Corti) contains inner and outer hair cells whose stereocilia transduce mechanical vibration into neural signaling. Sound-driven motion of cochlear membranes—particularly the basilar membrane—deflects stereocilia, leading to electrical responses and synaptic neurotransmission to spiral ganglion neurons, which relay information through the auditory nerve. Outer hair cells contribute to cochlear amplification and frequency selectivity, and their injury can therefore reduce sensitivity and degrade frequency tuning; damage to other elements of the transduction and synaptic machinery can further impair neural coding (Dewey et al. 2021).

Unlike many non-mammalian vertebrates, mammalian cochlear hair cells show extremely limited regenerative capacity, making permanent injury difficult to reverse (Wong et al. 2013). In contrast, hair-cell regeneration has been observed in other species and sensory systems, motivating interest in regenerative approaches—including stem cell–based strategies—as potential future therapies (Monroe et al. 2015). At present, however, multiple biological and translational barriers constrain clinically viable regeneration, and therefore NIHL management remains primarily focused on prevention and risk reduction, particularly in view of increasing exposure in modern environments.

Building on these mechanistic and regenerative constraints, a critical gap in NIHL research is a clear, intensity-dependent understanding of how synaptic elements at the IHC–spiral ganglion neuron (SGN) junction are injured and whether those changes track auditory function over time. Threshold shifts alone cannot fully capture “hidden” neural injury, and presynaptic ribbon counts by themselves may not distinguish persistent synapse loss from reversible disruption of pre–post alignment.

In this study, we aimed to determine whether IHC synaptic injury scales with pathological noise intensity and to define the longitudinal relationship between synaptic integrity and hearing function in a mouse model of NIHL. By comparing exposures associated with reversible versus persistent hearing deficits and relating structural synaptic readouts—including both ribbon abundance and pre–post synaptic pairing—to functional auditory measures, we aimed to identify synaptic metrics that most sensitively reflect preserved neural system. Clarifying these relationships provides an intensity-dependent map of synaptic vulnerability and recovery that can help interpret persistent auditory complaints despite partial threshold recovery and can inform the selection of practical endpoints for evaluating strategies designed to protect or restore cochlear synaptic connectivity after noise exposure.

## Materials and methods

### Animals and experiment design

Male mice of the C57BL/6N strain (8 weeks of age, Koatech, Daegu, Republic of Korea) were used, and their hearing ability was confirmed to be within normal range by auditory brainstem response measurements. Animals were randomly divided into four groups—exposed to white noise (2-20 kHz) 100 dB for 2 h or 4 h, and 110 dB for 2 h or 4 h to induce hearing loss. Two hours after exposed noise stimuli, measured hearing ability and monitoring at 1-week intervals during 3 weeks. All animal procedures were conducted in accordance with the Institutional Animal Care guidelines issued by the Committee of Animal Research of Kyungpook National University (2023-0160).

### Auditory brainstem response (ABR) measurement

To assess auditory function, we performed ABR measurements using an ABR workstation (System 3, Tucker Davis Technology (TDT), Inc., Alachua, FL, USA). All tests were conducted in a soundproof room. Briefly, prior to ABR measurement, animals were anesthetized by intramuscular injection of an alfaxan mixture and placed on a heating pad to maintain their body temperature at 37 °C. Mouse body temperature was monitored using a rectal thermometer. To record the ABRs, subcutaneous needle electrodes were inserted into the vertex (+ charge), mastoid (− e), and hind leg (ground). Acoustic stimuli, consisting of either a tone-burst stimulus with a 1-ms rise/fall time and a 5-ms plateau at frequencies of 8, 16, and 32 kHz or transient click stimuli, were applied monaurally through a speaker. The stimulus signals were generated by a SigGenRP and an RP2.1 real-time processor and then transmitted through a programmable attenuator (PA5, TDT), and an multi-field magnetic speaker (MF1, TDT). Stimuli were generated for 500 repetitions in 10-dB decrements starting from a 90-dB sound pressure level (SPL) to the acoustic threshold at every frequency. The phase of the stimulus was reversed upon each presentation to reduce the artefacts caused by repetitive stimuli.

### Antibodies

The primary antibodies used in this study are as follows: rabbit polyclonal anti-Myo7a (Proteus BioSciences, Ramona, CA, USA) and; mouse monoclonal anti-CtBP2 (BD biosciences, Franklin Lakes, NJ, USA); mouse monoclonal anti-GluR2 (Millipore Filter Corporation, Billerica, MA, USA). The secondary antibodies used for immunofluorescence were Alexa Fluor 488- or 568- conjugated goat anti-mouse IgG2a or IgG1, and 647-conjugated goat anti-rabbit IgG (Invitrogen, Waltham, MA, USA). All antibodies were diluted in 5% normal goat serum (NGS) with 0.1% Triton X-100 in 1 × PBS (PBS–Tx)..

### Immunostaining of cochlear whole mounts

For whole-mount immunostaining of the cochlea, inner ears were dissected out of the temporal bone, and a small opening was made in the oval window of the cochlea before fixation in 4% PFA in 1 × PBS (pH 7.4) overnight at 4 °C. After fixation, Reissner’s membrane, the lateral wall, and tectorial membranes were removed, and the remaining tissues were permeabilized by immersion in 5% Triton X-100 in PBS for 30 min, blocked with blocking solution containing 5% goat serum (Vector Laboratories Inc, Burlingame, CA, USA) in PBS for 1 h at RT, and stained overnight at 4 °C with primary antibodies. On the next day, the specimens were washed with PBS and incubated with secondary antibodies for 2 h at RT. The specimens were visualized using a Zeiss LSM 800 confocal microscope (Zeiss, Oberkochen, Germany). Zen 2012 software (Zeiss, Oberkochen, Germany) was used for image acquisition.

### Scanning electron microscopy

Cochlea was immediately isolated from the euthanized mice and perfused carefully through the round window using 2.5% glutaraldehyde and 2% paraformaldehyde in 0.1 M sodium cacodylate buffer (pH 7.4) after a hole was made at the top of the cochlea. The perfused cochleas were immersed in the same fixation mixture for 1 h at room temperature. The lateral wall, Reissner’s membrane and the tectorial membrane were removed via dissection under a dissecting microscope and fixed overnight at 4 °C in 0.1 M sodium cacodylate buffer (pH 7.4) containing 2.5% glutaraldehyde, 2 mM calcium chloride and 3.5% sucrose. After fixation, the dissected specimens were rinsed three times for 20 min at 4 °C with 0.1 M sodium cacodylate buffer containing 2 mM calcium chloride. The specimens were treated post-fixation using the osmium tetroxide (OsO_4_)- thiocarbohydrazide. Briefly, specimens were immersed in 1% OsO_4_ for 1 h at 4 °C and then placed in saturated thiocarbohydrazide for 20 min at room temperature. This specimen treatment was repeated three times at room temperature. The specimens were then dehydrated using a graded series of ethanol solutions, dried using a critical point drier (HCP-2, Hitachi), affixed on a stub and coated with platinum using a sputter coater (E1030, Hitachi). The coated specimens were mounted on a stub holder and viewed using a coldfield emission scanning electron microscope (S-4300, Hitachi) operated at 15 kV.

### Statistical analyses

Statistical analyses were performed using 2-tailed Student's *t*-test, with *P* < 0.05 being considered statistically significant. Data was analyzed by comparing treated and untreated contralateral sides or by comparing treated and control mice.

## Results

### Hearing function change pattern according to noise stimulation

**Fig. 1:**
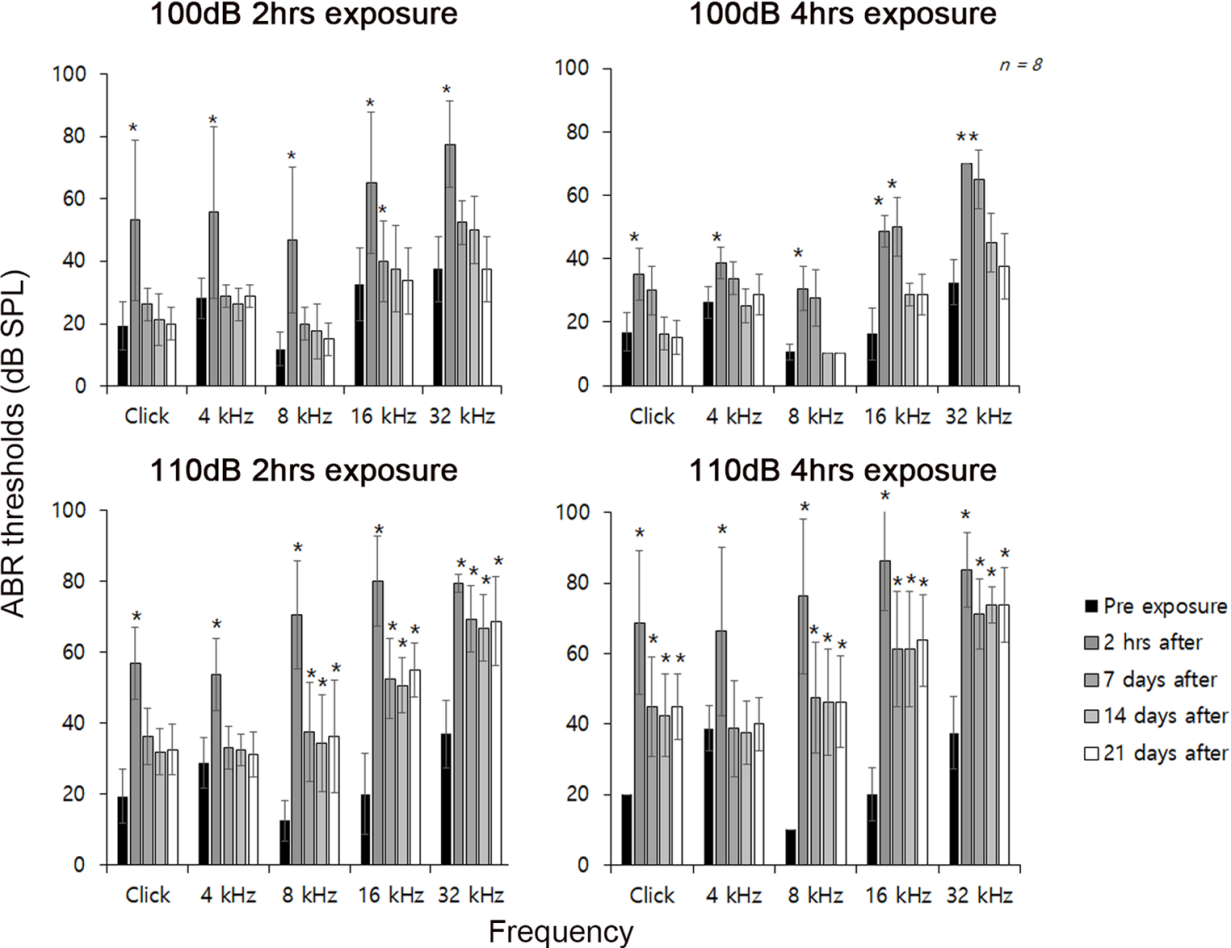
ABR measurement of noise-induced mice model. ABR thresholds of average click stimuli and 4-, 8-, 16-, and 32-kHz tone-burst stimuli in 4 experiment group—exposed to white noise 100 dB for 2 h or 4 h, and 110 dB for 2 h or 4 h to induce hearing loss. Hearing was measured 2 h after noise stimulation, and then hearing was checked for 3 weeks at intervals of 1 week. (*P* > 0.05; *n* = 8)

To determine the temporal thresholds shift (TTS) model and permanent thresholds shift (PTS) model, different noise level of white noise (2–20 kHz) was exposed to mice on 8 weeks. Baseline ABR thresholds were measured at 8 weeks of age prior to noise exposure to confirm normal hearing. The mice stimulated with noise stimulation for 100 dB for 2 h showed an increase in the value of the hearing thresholds of about 30 dB at 2 h after the noise stimulation, and the mice began to recover by day 7 post-exposurethe noise stimulation. These aspects were sustained and completely restored to normal hearing 21 days after noise stimulation. Similarly, mice that stimulated 100 dB for 4 h showed an increase in hearing threshold value of more than 30 dB immediately after noise stimulation, but did not fully recover to the normal level in the 16 and 32 kHz frequency region (Fig. 1).

Exposure to 110 dB for 2 h or 4 h produced an ~ 50 dB threshold shift at 2 h post-exposure. Hearing gradually recovered after 7 days of noise stimulation, but it did not completely return to normal hearing, and the difference was most noticeable in the mice stimulated with 110 dB for 4 h (Fig. 1). Therefore, we defined mice exposed to 100 dB for 2 h as a TTS model and mice exposed to 110 dB for 4 h as a PTS model.

**Fig. 2:**
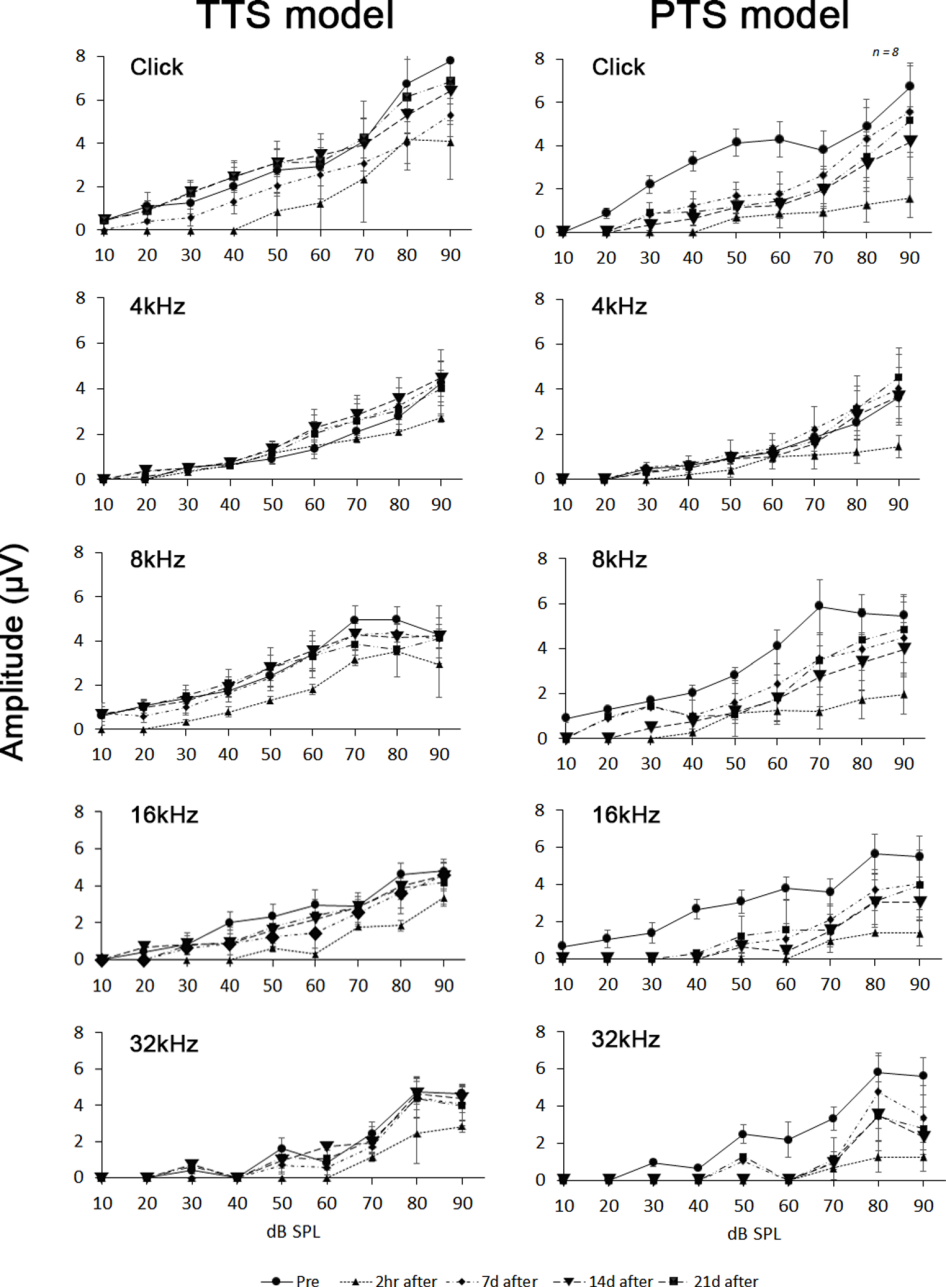
Amplitude analysis in TTS model and PTS model. Average peak amplitudes for wave-I was measured of click stimuli and 4-, 8-, 16-, and 32-kHz tone-burst stimuli in TTS model and PTS model. It shows the appearance of changes measured at weekly intervals (*n* = 8)

The most commonly reported biomarker of synaptopathic noise-induced trauma is a marked reduction in ABR wave-I amplitude and increase of latency at suprathreshold stimulus levels. Following the TTS model noise exposure, there were no permanent changes in ABR wave-I amplitude 21 days post exposure; all of the observed changes were temporary and reversible. In contrast, there was a reduction in ABR wave-I amplitude 21 days after the PTS model noise exposure at all five test frequencies (click, 4, 8, 16, and 32 kHz) that failed to recover at all frequency 21 days post exposure test. Post-mortem analysis showed that after 2 h of baseline and noise, there was a significant difference in ABR wave-I amplitude after 21 days of baseline and noise, and the ABR amplitude did not return to baseline levels after 21 days of exposure. Differences in ABR amplitude were found at baseline and presentation levels of 60, 70, 80 and 90 dB SPL at 16, 24 and 32 kHz for 21 days after exposure, but differences in amplitude at stimulation levels of 20 and 30 dB SPL were not found. (Fig. 2).

**Fig. 3:**
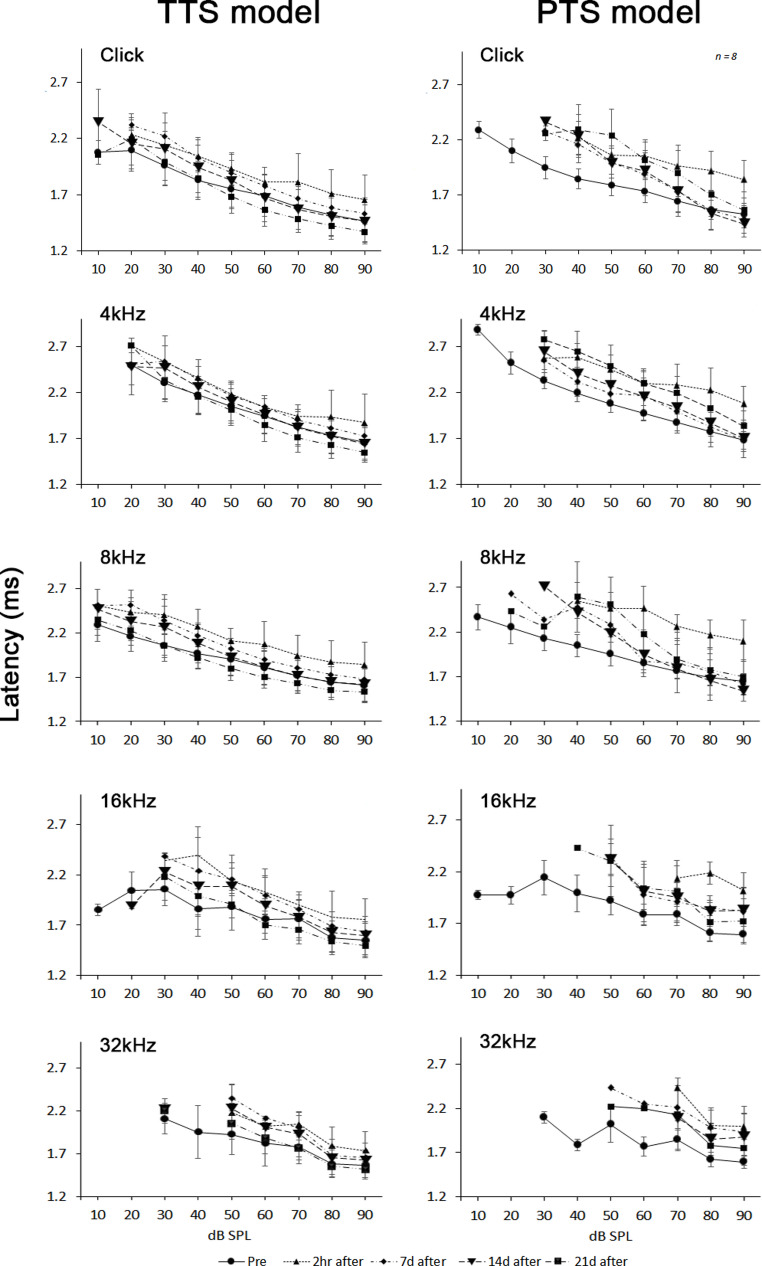
Latency analysis in TTS model and PTS model. Average peak latencies for wave-I was measured of click stimuli and 4-, 8-, 16-, and 32-kHz tone-burst stimuli in TTS model and PTS model. It shows the appearance of changes measured at weekly intervals (*n* = 8)

These results also appeared when measuring the latency. In the TTS model, the increased latency values returned to normal after 21 days, whereas in the PTS model, the increased latency after noise stimulation did not return normally even after 21 days (Fig. 3).

### Hair cell morphology in noise-induced hearing loss mouse model

The hair cell plays a role in first receiving auditory stimulation and converting vibrational energy into an electric signal. Therefore, we tried to investigate the damage to the hair cells that would be primarily damaged by noise stimulation.

**Fig. 4:**
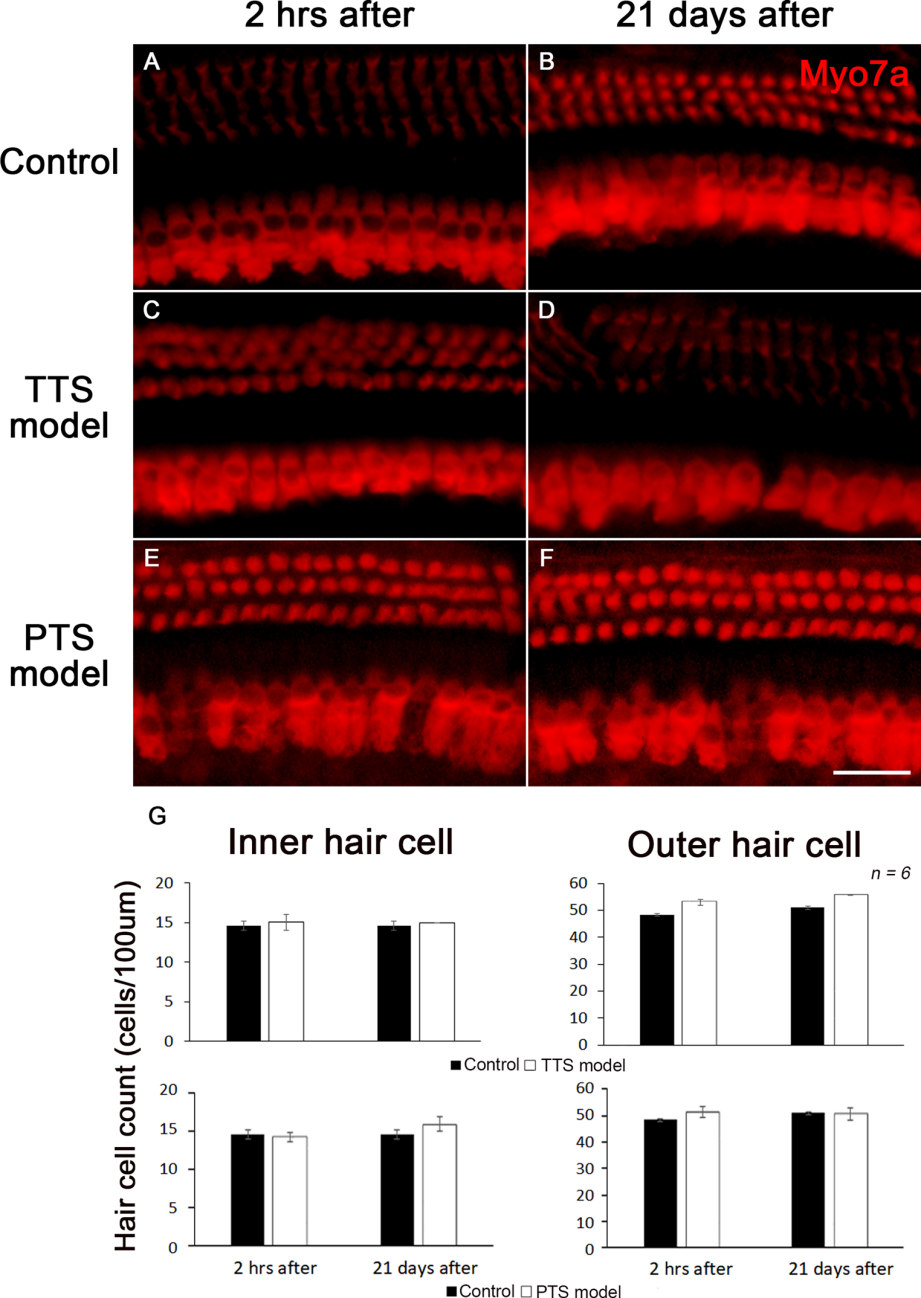
Immunohistochemical analysis of hair cell damage in the noise-induced mouse model. Immunohistochemical analysis was performed at 2 h after and 21 days after noise exposed in TTS and PTS model. Hair cells were labeled with Myo7a (red, **A**–**F**). The graph shows the number of each inner hair cell and outer hair cell in TTS and PTS model (**G**). Scale bar represent 25 μm. Data are expressed as mean ± SD

Immunohistochemical analysis was performed to analyze hair cells in the inner ears of noise-induced hearing loss mouse model. We counted the number of hair cells dyed with Myo7a to see if they were affected by noise. As a result, in the TTS group, there were no differences between 14.7 and 15 inner hair cells before and after each 100um noise, and there were no differences in the outer hair cells to 48.2 and 50. Surprisingly, these results were the same in the PTS group. Before and after noise stimulation of the PTS group, there were 14.8 and 14.7 inner hair cells, respectively, and 47.4 and 50 outer hair cells, respectively, which showed no effect on the number of hair cells. Likewise, the overall shape of the hair cell did not show any significant damage (Fig. 4).

### Ultrastructure of the stereociliary bundle in auditory hair cells of TTS and PTS model

**Fig. 5:**
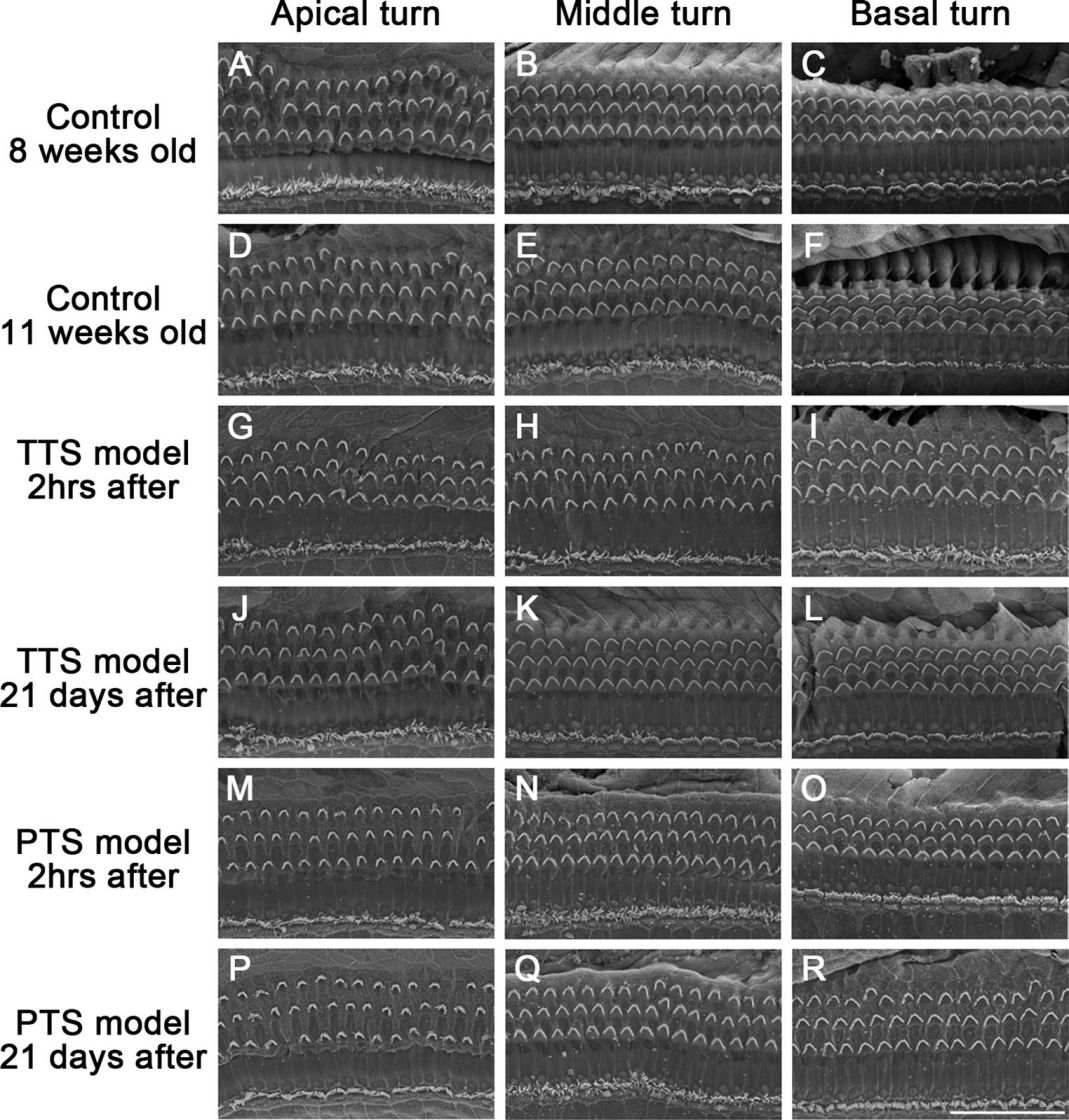
Ultrastructure of the stereociliary bundles. TTS and PTS mouse model were euthanized at noise exposed 2 h after and 21 days after for performing SEM. Control group (**A**–**F**) TTS group (**G**–**L**) and PTS group (**M**–**R**). Scale bar represent 30 μm

Ultrastructure of hair cells of TTS and PTS model were determined by performing SEM at the noise exposure 2 h after and 21 days after to identify of stereociliary bundle. The stereociliary bundle is generally arranged in 3 rows of outer hair cells in a “V” or “W” shape and 1 row of inner hair cells in a linear form (Fig. 5A–F). Stereociliary degeneration was not observed in all the cochlear turns of TTS and PTS model. Inner hair cells of TTS and PTS model showed a normally linear arrangement of stereocilia; and also, cell degeneration was not observed all cochlear turns (Fig. 5G–R). Most stereocilia in the outer hair cells of TTS and PTS model were not degenerated, and the remaining stereocilia were perfect arranged. Moreover, hair cells in the inner ears of TTS and PTS model showed exactly 3 rows of outer hair cells and 1 row of inner hair cells. These results indicated that TTS and PTS model were not induced morphological defects in both stereocilia and hair cells.

### Ribbon synapses are degenerated in the inner ears of noise-induced hearing loss mouse model

**Fig. 6:**
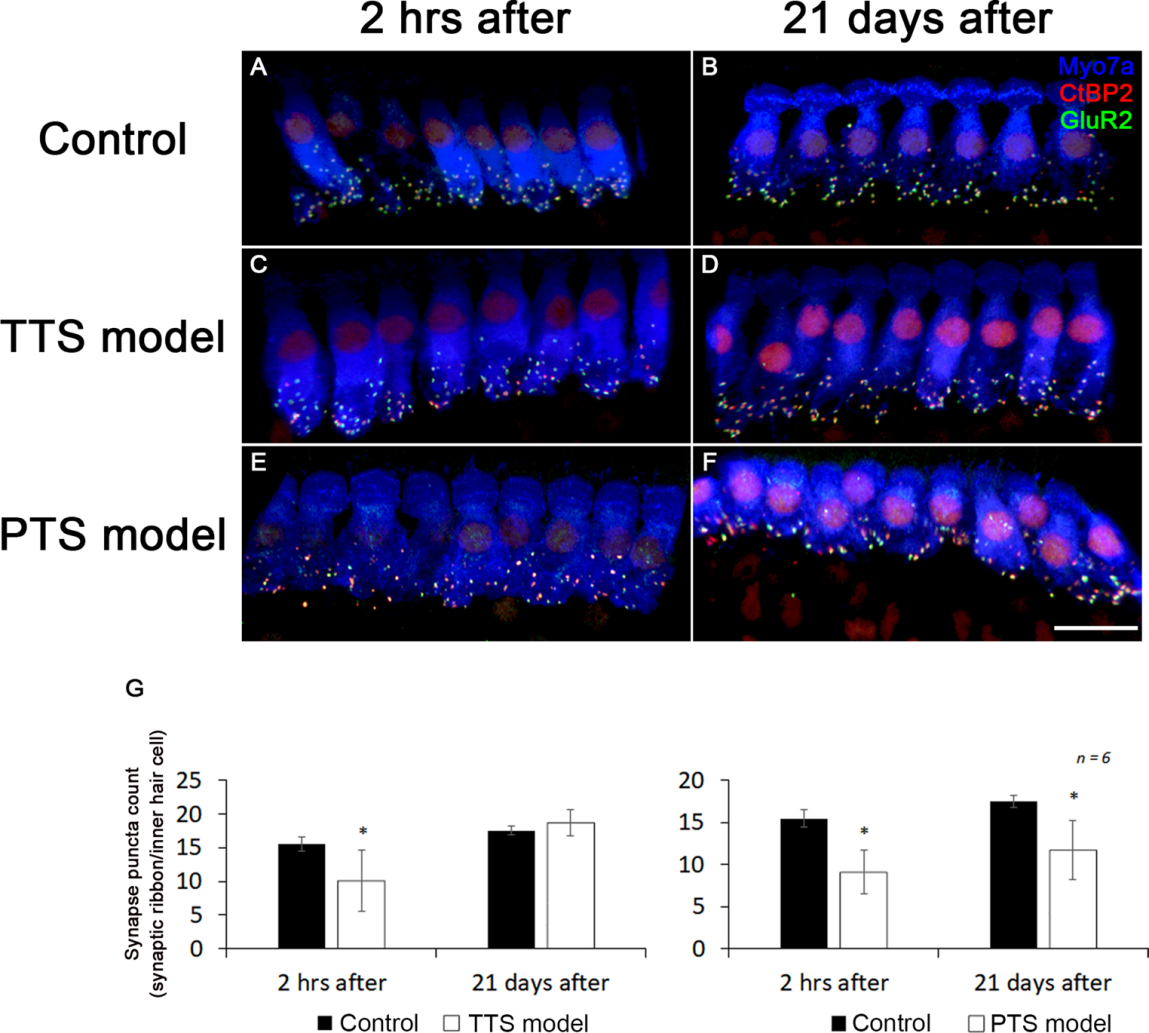
Degeneration of ribbon synapse in the organ of Corti of noise-induced hearing loss mouse model. Whole-mount immunostaining of the organ of Corti of TTS and PTS model. Hair cells were labeled with Myo7a (blue), pre-synapse was labeled with CtBP2 (red), and post-synapse was labeled with GluR2 (green). Control group (**A**, **B**) TTS group (**C**, **D**). PTS group (**E**, **F**). The graph shows the number of ribbon synapse in inner hair cells (**G**). Scale bar represent 10 μm. (*P < 0.05, *n* = 6). Data are expressed as mean ± SD

To determine in noise-induced hearing loss mouse model of IHC synaptic connections, we first quantified ribbons at 2 h, 21 days after TTS and PTS model. Exposure to the noise significantly reduced synaptic ribbon counts by 45% 2 h after the exposure at 16 kHz region. The ribbon loss remained stable and significantly lower than those of unexposed controls for at least 21 days, the latest time measured (Fig. 6). We also identified functional IHC synapses as juxtaposed presynaptic ribbons and postsynaptic glutamate receptors examined 2 h and 21 days after the TTS and PTS model. In agreement with the loss of ribbons, the synapses were also reduced at TTS and PTS model. Furthermore, PTS noise exposure induced a greater amount of synaptic ribbon loss at the high-frequency regions after the exposure evaluated at 2 h, which also did not recover 21 days after the exposure (Fig. 6G). In addition, TTS noise exposure reduced functional IHC synapses at 2 h after, but it recovered at 21 days after exposure. Further analysis revealed that the differences between the control group and 2 h after PTS groups at 16 kHz were significant. The loss of synapses at PTS group was less than those lost at 2 h after a TTS noise exposure. Furthermore, to confirm whether synapses with juxtaposed CtBP2 and GluA2 indeed represented functional synapses, we conducted regression analysis that showed a correlation between synapse number and auditory function. Two hour after exposure, CtBP2-immunolabeled synaptic ribbons of IHCs decreased on surface preparations in the 16 kHz region. Since ABR wave-I amplitudes indicate the activity of the auditory nerve, we measured the amplitude of ABR wave-I. Twenty one days after exposure to TTS model, the noise-induced decrease in wave-I amplitudes was significantly elevated at normal amplitude. These results show that changes in amplitude and ribbon synapse are correlated.

## Discussion

Our data support the idea that cochlear pathology after acoustic overexposure is not limited to OHC damage, but extends to IHC synaptopathy in an exposure-intensity–dependent manner. In our paradigm, 100 dB noise for 2 h and 110 dB noise for 4 h produced graded effects on IHC ribbon synapses and on the number of functionally aligned synaptic contacts. To quantify functional IHC–spiral ganglion neuron (SGN) synapses, we measured closely apposed pre- and postsynaptic markers, defining an intact contact as a presynaptic ribbon punctum (CtBP2 immunolabel) that was juxtaposed to a postsynaptic AMPA receptor punctum (GluA2 immunolabel). Using this operational definition, the number of CtBP2/GluA2 apposed pairs (i.e., putative functional synapses) closely tracked preserved hearing function, indicating that residual auditory performance is better explained by the amount of remaining functional connectivity than by ribbon counts alone.

Longitudinally, noise-induced loss of synaptic ribbons appeared largely stable across the post-exposure intervals examined, suggesting that ribbon disappearance itself is not strongly progressive over time under these conditions. In contrast, the early post-exposure reduction in functional synapses was disproportionately larger than the reduction in ribbons, implying that immediately after noise trauma a substantial fraction of surviving ribbons may be transiently “uncoupled” from their postsynaptic specializations. Importantly, the subsequent increase in apposed CtBP2/GluA2 pairs toward the level predicted by the remaining ribbon pool is consistent with the possibility that a subset of initially misaligned or disconnected synaptic elements can re-establish functional alignment during recovery, approaching the number of ribbons that persisted after the initial insult (Liberman et al. 2015; Wan et al. 2014). In line with this interpretation, the increased variability we observed in ribbon-associated measures at early time points (e.g., 2 h after exposure) is compatible with prior observations that pre–post misregistration can yield morphologically detectable but functionally ineffective synaptic sites (Liberman et al. 2015).

These findings integrate well with current mechanistic frameworks for noise-induced hearing loss. Beyond direct hair-cell injury, synaptic vulnerability at the IHC–SGN junction is now recognized as a key contributor to sustained auditory deficits. SGN afferents can be broadly classified by spontaneous firing and threshold characteristics: high-spontaneous-rate fibers with low thresholds encode low-level sounds, whereas low-spontaneous-rate fibers typically require higher sound levels to respond. Preferential impairment of high-threshold/low-spontaneous-rate fibers has been proposed to produce a “hidden” deficit—where thresholds may partially recover while suprathreshold processing (including speech perception in background noise) remains compromised—an outcome often associated with noise exposure (Kohrman et al. 2020; Furman et al. 2013).

Multiple injury pathways may converge on this synaptic phenotype. Classic models emphasize excessive mechanical stress within the organ of Corti, including disruption of micromechanical coupling in stereocilia; partial restoration of these structures could explain transient threshold shifts, whereas repeated or severe exposures promote secondary cellular pathology that compromises neural terminals and supporting elements (Han et al. 2020; Hu et al. 2020; Kurabi et al. 2017). Metabolic overload and oxidative/nitrosative stress provide an additional layer of explanation: loud sound can elevate mitochondrial demand, increasing reactive oxygen and nitrogen species and triggering downstream cascades that include inflammatory signaling (e.g., IL-6, TNF-α) and apoptotic pathways such as caspase activation (Han et al. 2018; Sai et al. 2022; Wu et al. 2020). Notably, reactive-species–linked injury has been suggested to evolve over an extended time window in some settings, implying that post-exposure interventions targeting oxidative stress and inflammation could remain effective beyond the immediate exposure period. Vascular dysregulation may further amplify these processes, as microcirculatory disturbance and ischemic stress can promote oxidative injury and potentially create self-reinforcing feedback that exacerbates cochlear damage (Campbell et al. 2023; Teraoka et al. 2024; Yamashita et al. 2005). Finally, excessive glutamate release at the IHC synapse (excitotoxic stress), disturbed calcium homeostasis, and downstream signaling (including calcineurin and phospholipase A2 activation) have been proposed as additional contributors to acute synaptic dysfunction and longer-term degeneration. Together, these pathways highlight multiple potential therapeutic entry points and may also help explain inter-individual differences in susceptibility to identical acoustic insults through genetic variability in stress-response and repair mechanisms.

## Data Availability

The data that support the findings of this study are available from the corresponding author, upon reasonable request.
