# Correction to: Cochlear synaptic vulnerability scales with pathological noise intensity in a noise-induced hearing loss model

**DOI:** 10.1007/s13258-026-01770-y

**Published:** 2026-06-16

**Authors:** Byeonghyeon Lee, Tae-Jun Kwon, Hyun Tae Kim, Un-Kyung Kim

**Affiliations:** 1 New Drug Development Center, Daegu-Gyeongbuk Medical Innovation Foundation (K-MEDI hub), Daegu, 41061 Republic of Korea; 2 Preclinical Research Center, Daegu-Gyeongbuk Medical Innovation Foundation (K-MEDI hub), Daegu, 41061 Republic of Korea; 3 Bio-Medical Research Institute, Kyungpook National University Hospital, Daegu, 41940 Republic of Korea; 4 Department of Biology, College of Natural Sciences, Kyungpook National University, Daegu, 41566 Republic of Korea; 5 School of Life Sciences, BK21 Plus KNU Creative BioResearch Group, Kyungpook National University, Daegu, 41566 Republic of Korea; 6 Adcanced Bio-Resource Research Center, Kyungpook National University, Daegu, 41566 Republic of Korea

**Correction to: Genes & Genomics** 10.1007/s13258-026-01748-w

In the original publication of this article, an error was identified in Figure 4, specifically in panels E and F. During the preparation of the figure, multiple image datasets were processed as part of blinded hair cell counting analyses, in which images from different experimental groups and time points were handled independently to minimize bias. During the subsequent organization and compilation of these datasets into the final figure, an inadvertent image mix-up occurred. As a result, the image presented in Figure 4E (intended to represent the PTS model at 2 h after noise exposure) was mistakenly replaced with an image corresponding to Figure 4F (21 days after noise exposure), leading to an unintended duplication between the two panels.

To ensure transparency and clarify the source of the corrected data, the raw image datasets corresponding to each experimental group in Figure 4 are provided below. The specific regions used in the published figure are indicated by white boxes. Each dataset consists of multiple segmented image fields acquired from a single cochlea, reflecting the fields used for quantitative hair cell analysis.

**1. Figure 4A** (Control 2 h after) raw data set:

**Figure:**
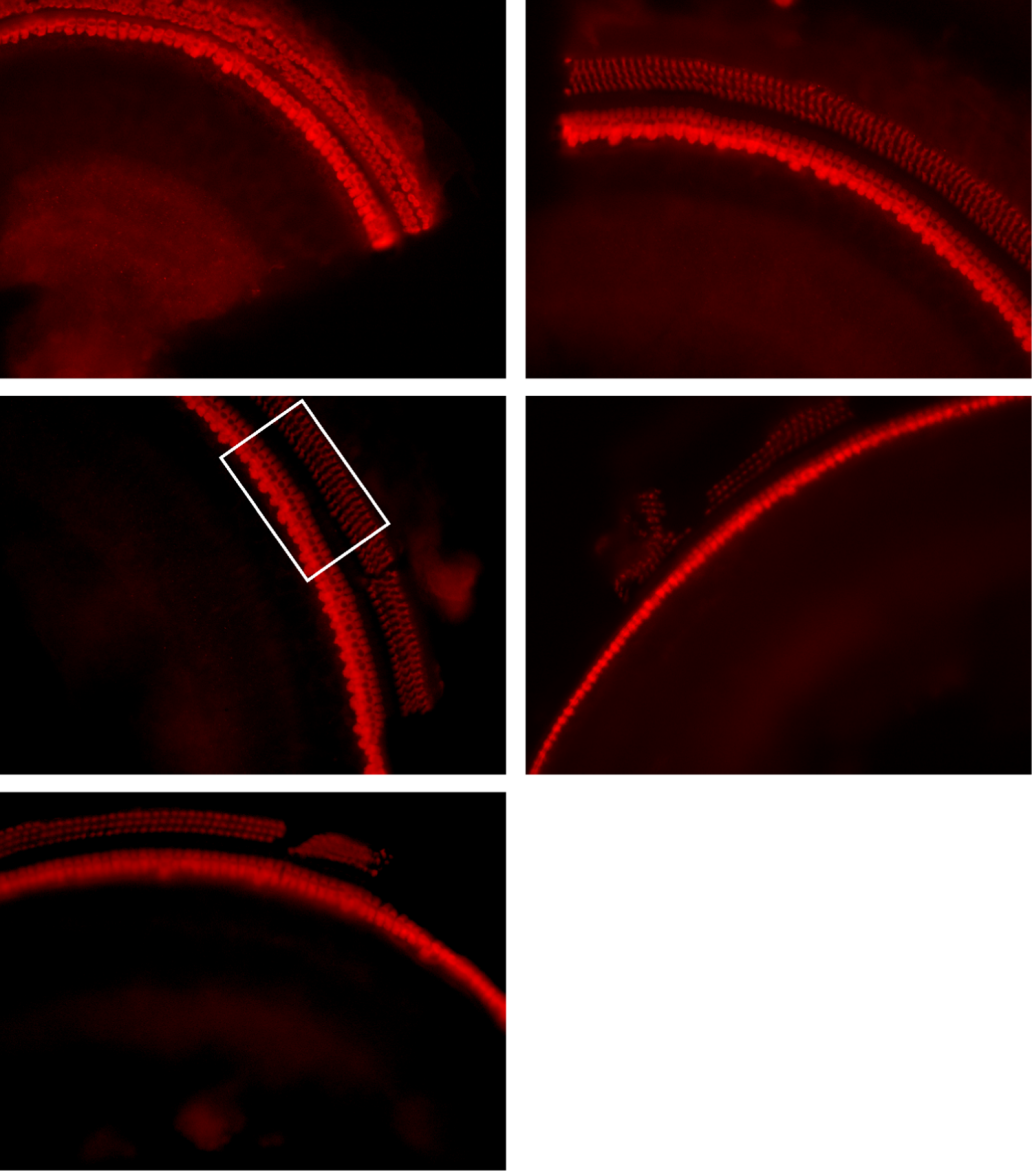

**2. Figure 4B** (Control 21 days after) raw data set:

**Figure:**
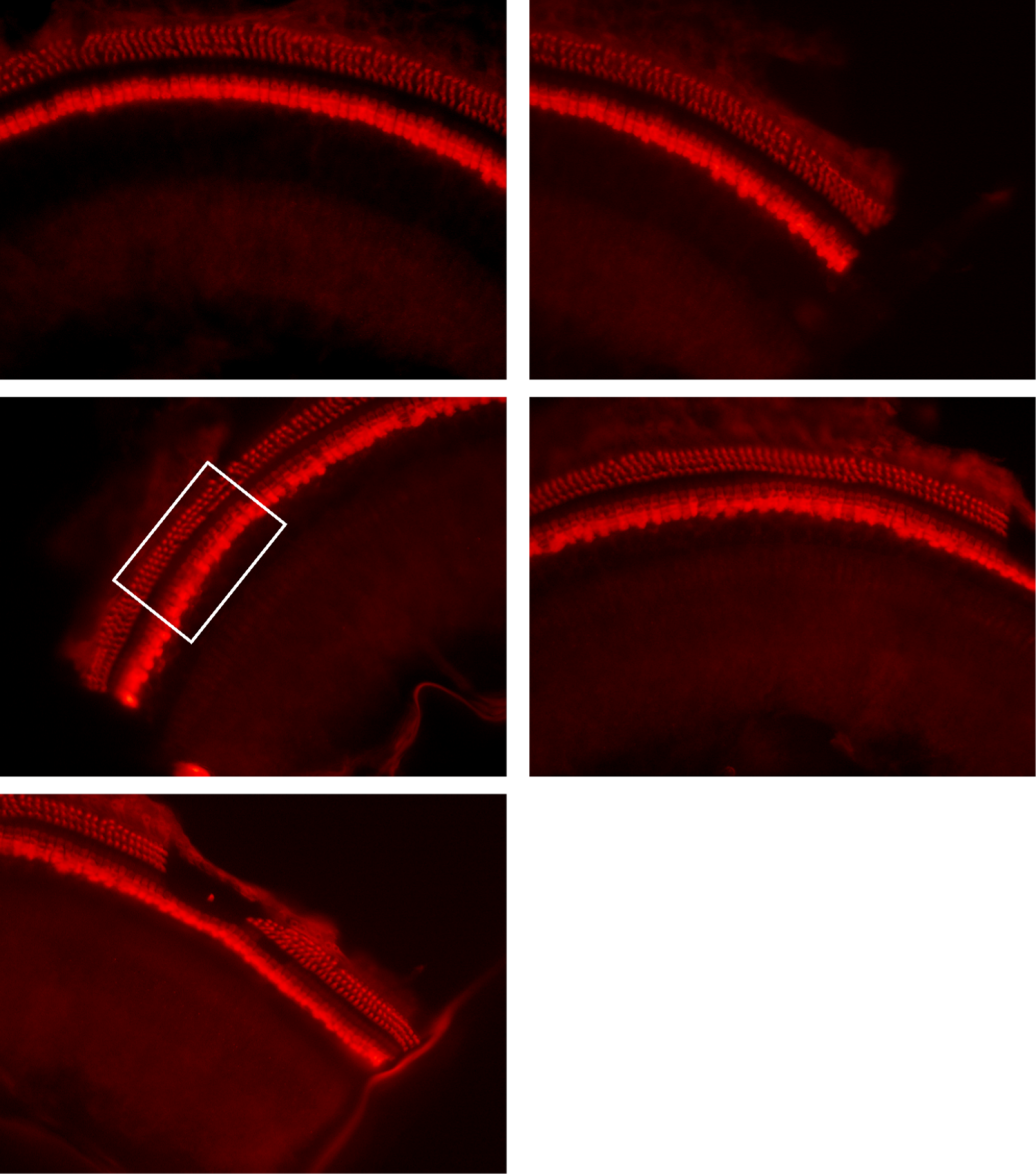

**3. Figure 4C** (TTS model 2 h after) raw data set:

**Figure:**
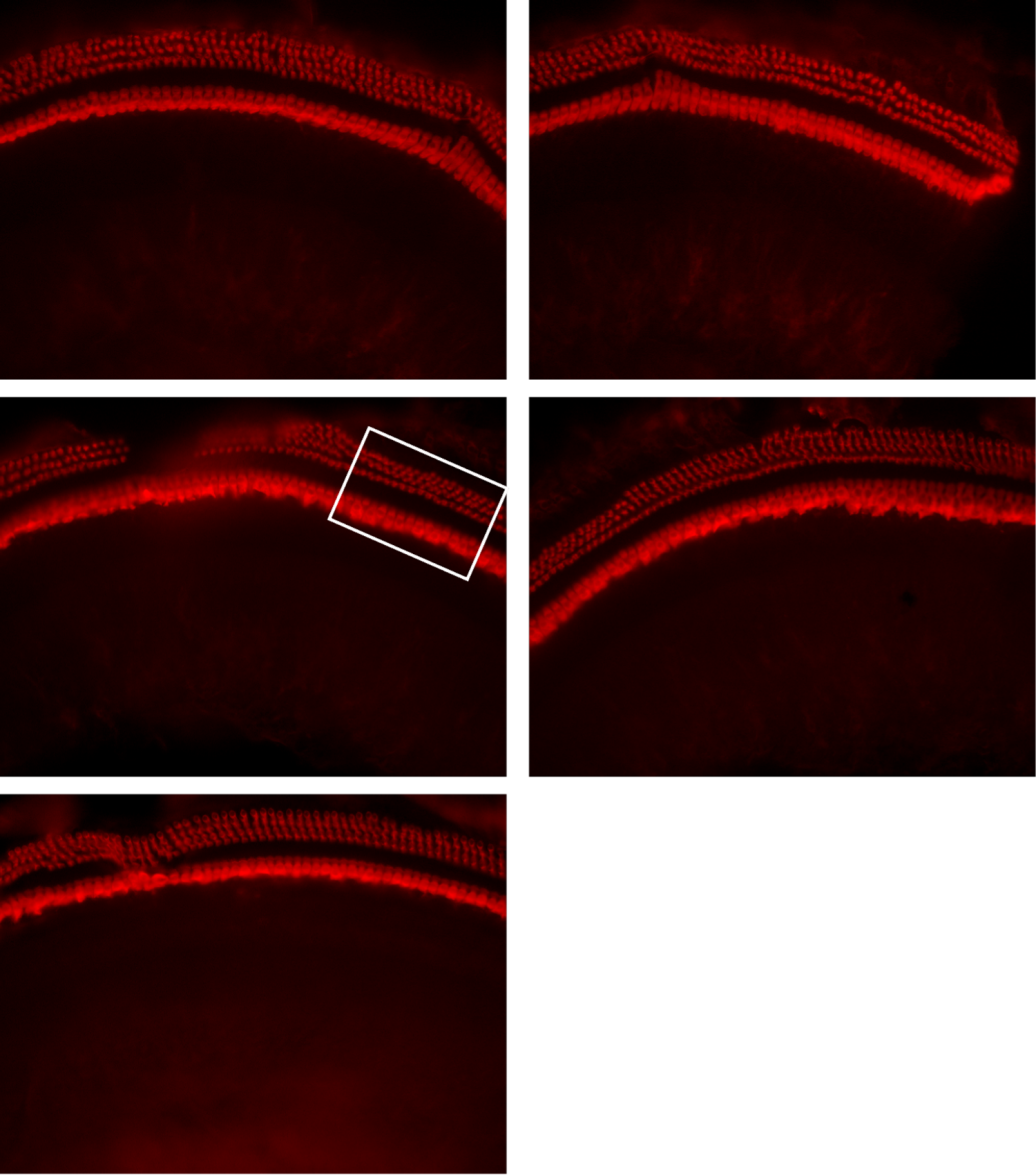

**4. Figure 4D** (TTS model 21 days after) raw data set:

**Figure:**
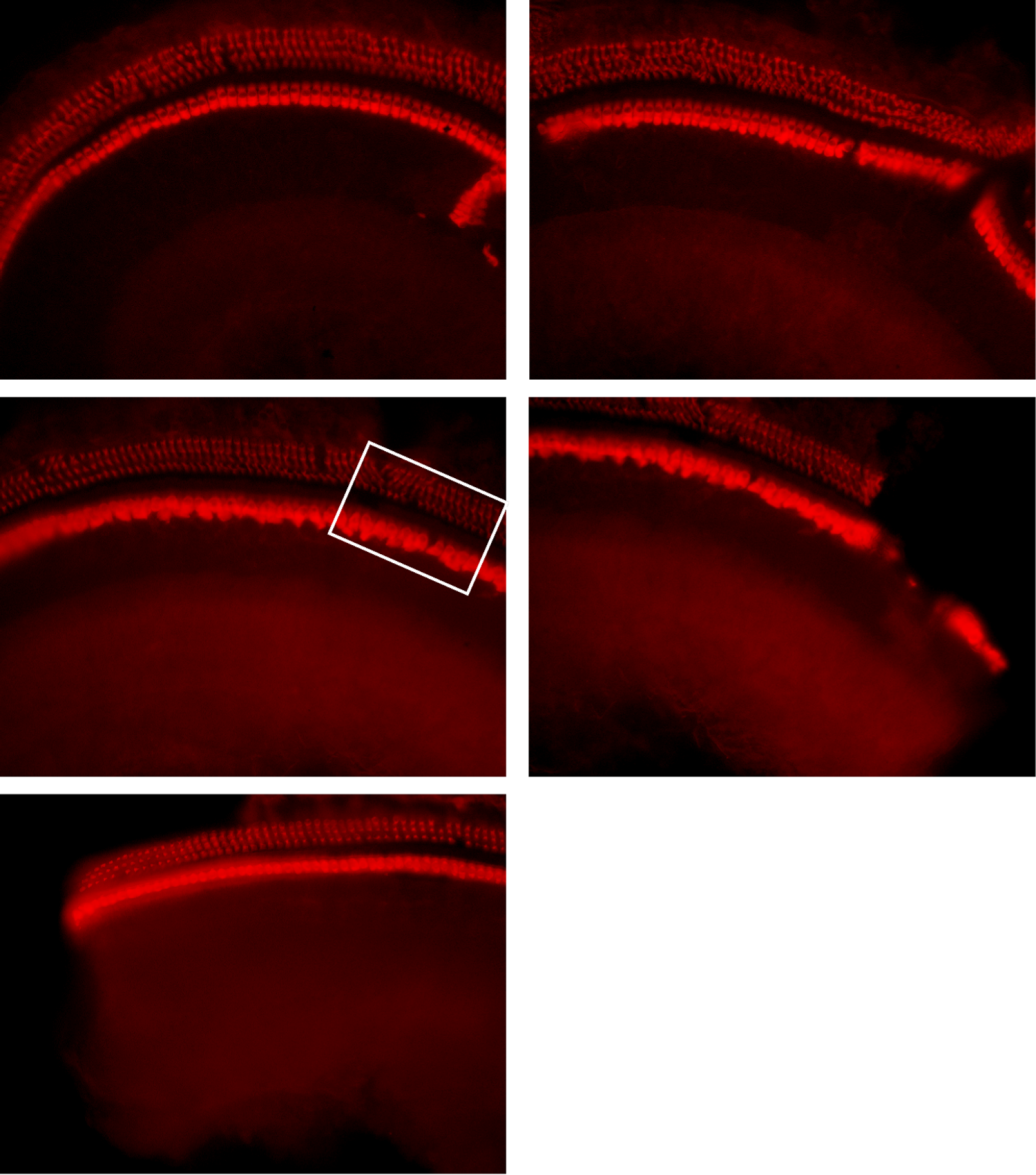

**5. Figure 4E** (PTS model 2 h after) raw data set:

**Figure:**
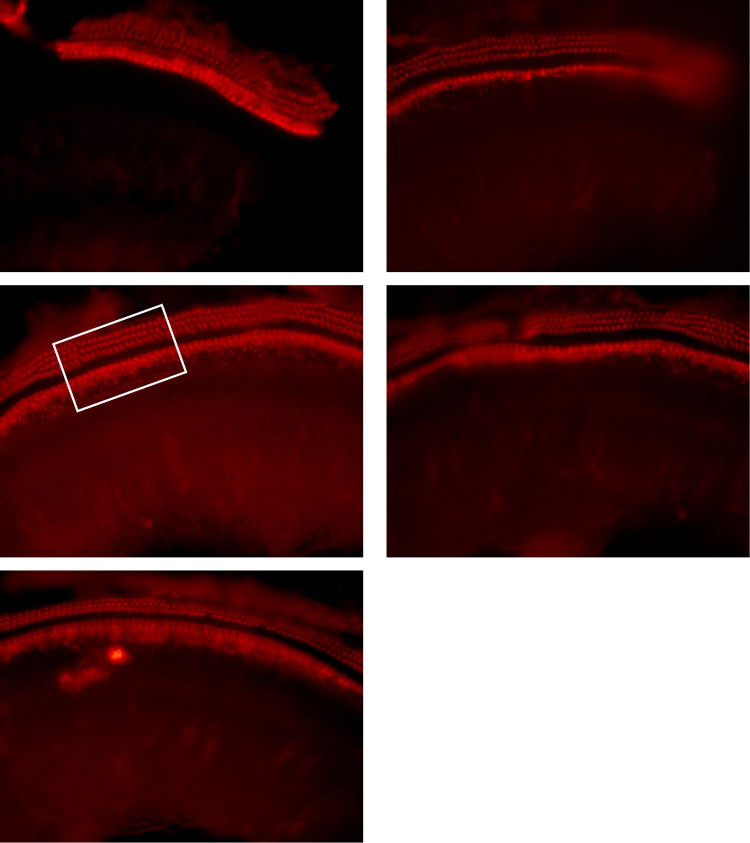

**6. Figure 4F** (PTS model 21 days after) raw data set

**Figure:**
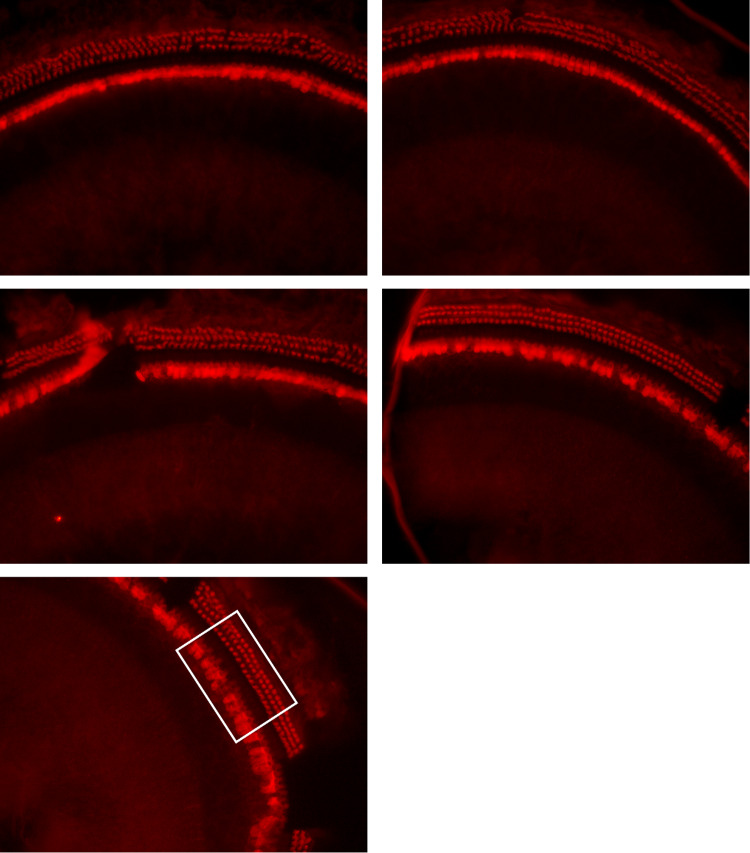

The corrected Figure 4 is provided below.

**Fig. 4:**
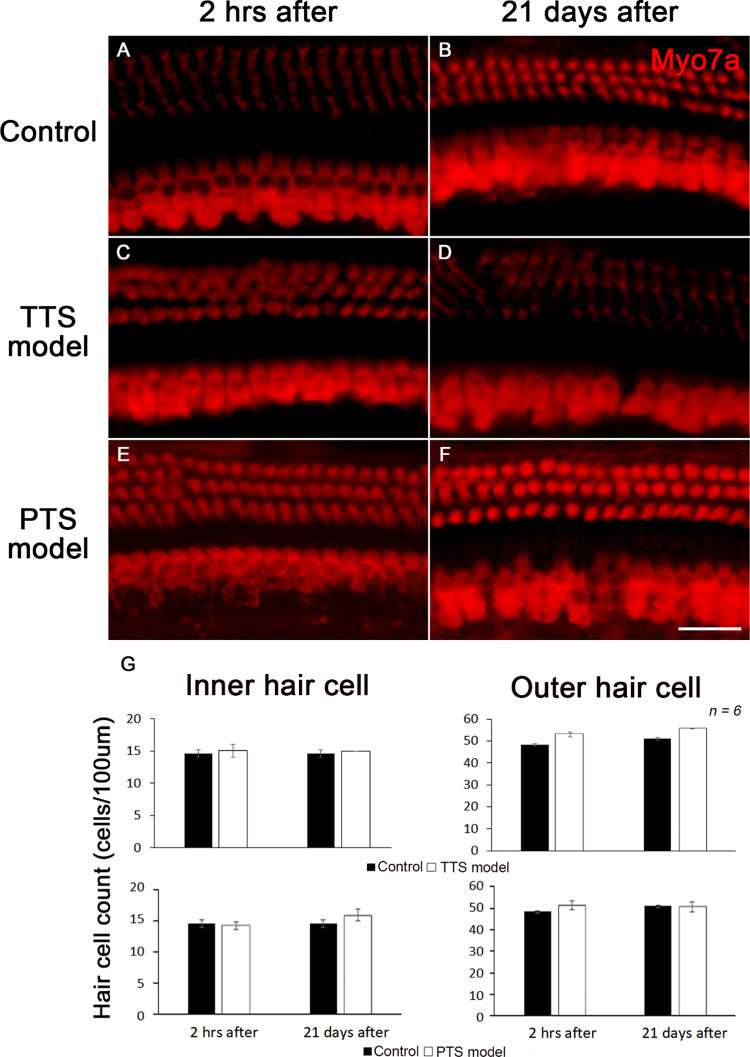
Immunohistochemicalanalysis of hair cell damage inthe noise-induced mouse model.Immunohistochemical analysiswas performed at 2 h after and21 days after noise exposed inTTS and PTS model. Hair cellswere labeled with Myo7a (red,**A**–**F**). The graph shows the number of each inner hair cell andouter hair cell in TTS and PTSmodel (**G**). Scale bar represent25 μm. Data are expressed asmean ± SD

Importantly, this error was confined solely to the figure assembly process and did not arise from any inaccuracies in the underlying raw data or analytical procedures. Accordingly, the quantitative analyses, results, and overall conclusions of the study remain unchanged and are not affected by this correction.

The authors sincerely apologize for this oversight and for any confusion or inconvenience it may have caused to the readers, reviewers, and editors. We greatly appreciate the opportunity to correct the record and remain committed to maintaining the highest standards of accuracy and transparency in our work.

